# Contact-Hardening Behavior of Calcium Silicate Hydrate Powders

**DOI:** 10.3390/ma11122367

**Published:** 2018-11-25

**Authors:** Shuping Wang, Xiaoqin Peng, Luping Tang, Chunpeng Cao, Lu Zeng

**Affiliations:** 1 College of Materials Science and Engineering, Chongqing University, Chongqing 400045, China; pxq01@cqu.edu.cn (X.P.); zool@foxmail.com (L.Z.); 2 Department of Architecture and Civil Engineering, Chalmers University of Technology, 412 96 Gothenburg, Sweden; tang.luping@chalmers.se; 3 Yunnan Institute of Building Research, Kunming 650223, Yunnan, China; xyxljyl@sina.com

**Keywords:** calcium silicate hydrate, compression, compacts, point contact, interparticulate bonding

## Abstract

Calcium silicate hydrate (C-S-H) synthesized by a hydrothermal process from lime and siliceous materials was oven-dried and compressed into compacts. The microstructure and compaction properties of the resulting powders were characterized. The results show that the powders containing an amorphous structure become hardened compacts immediately after compression. Compacts with high strength but a relatively lower bulk density were produced. Amorphous C-S-H plays a key role in the bonding formation during powder compaction. According to the Heckel plots, particle rearrangement and plastic deformation were involved in the compaction of C-S-H powders. Point contact between C-S-H particles due to particle rearrangement dominates at a low compression pressure (i.e., <20 MPa). Van der Waals forces and hydrogen bonding are the main bonding types. Plastic deformation occurs at a higher compression pressure (i.e., >60 MPa), which results in surface contact. Consequently, a solid bridge forms, and the strength of compacts increases rapidly. These findings provide novel insight into the utilization of materials containing amorphous calcium silicate hydrate.

## 1. Introduction

**Figure 1:**
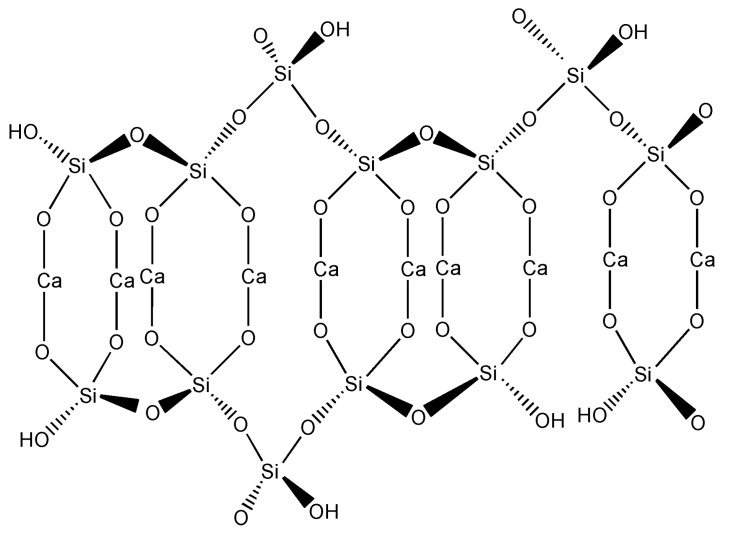
C-S-H structure with probable positions of H atoms and suggested modification by elimination of bridging tetrahedron in silicate chain (according to [5]).

Calcium silicate hydrate, or C-S-H (C=CaO, S=SiO_2_ and H=H_2_O), is the main hydration product of cementitious material with Ca/Si ratios from 0.6 to 2.3 [1]. It is believed to have a disordered layer structure based on silicate chains with calcium sheets in between. The silicate chain is a repeated structure of three tetrahedrons, two of which are paired connected by a bridging tetrahedron (Figure 1). The paired oxygen atoms in the silicate tetrahedron are connected with a CaO back-bone. In addition, some oxygen ions can be replaced by hydrogen (-OH) groups due to the omission of the bridging tetrahedron. Water molecules and Ca^2+^ can be observed in the interlayer space [2]. Due to the liquid (water) and solid (silicate tetrahedrons and calcium sheet) phases in C-S-H, it is considered a major contributor to the mechanical and physical properties of cement and concrete. For example, Pellenq and co-authors [3] suggested that the cohesion force was attributed to the electrostatic nature between C-S-H particles via Monte Carlo simulation. They also believed that the interaction of Van der Waals force, capillary force, and ionic-covalent bonding between individual C-S-H layers or C-S-H layer stacks plays a role in the hardening behavior [3]. The hardening property of C-S-H is developed during the hydration process when the cement reacts with water. Interestingly, researchers found that C-S-H powders can be compacted and hardened by compression at certain pressures, causing no chemical reaction to occur [4].

The compression method has been widely applied in the practical industries for several decades, for example, in pharmaceutics [6], biomass fuel transportation [7], and ceramic and powder metallurgical industries [8]. Compression of cementitious materials was initially applied to study the isothermal sorption and microstructure of rigid porous compacts in the 1960s and 1970s [9,10]. Beaudoin compressed a mixture of C-S-H and Ca(OH)_2_ powder at pressures from 136 to 680 MPa to investigate the role of Ca(OH)_2_ in the mechanical properties of the compacts [11]. He found that the presence of Ca(OH)_2_ could lower the fracture strength of the specimen. Jennings [12] used this method to produce low-density, hydraulic bonded composite articles, and Stemmermann et al. [13] prepared bonded components with high tensile strength by compaction of nanocrystalline C-S-H phases. Lin et al. [14] studied the dynamic mechanical behavior of calcium silicate hydrate under shock compression loading, and reported that collapse and compaction occurred in C-S-H depending on the particle velocity. Recent research also showed that the drying conditions on C-S-H affect the bonding between particles and on the mechanical properties of the compacts [15]. An advantage of this approach is that specimens with high strength can be obtained after a few minutes of compression rather than a long curing time. However, it is unclear why these powders can be hardened by compression. Glukhovsky et al. [16] investigated the hardening properties of compacts prepared from the powders containing C-S-H. They showed that the disordered C-S-H plays a key role in the hardening of the compacts, and a rigid structure can be formed when the particles come into contact, i.e., so called “contact-hardening”; however, the investigation could not be continued due to Glukhovsky’s demise. The compression properties of calcium silicate hydrate, and the contact-hardening mechanism of C-S-H powders, remain unclear.

In this study, the contact-hardening behavior of calcium silicate hydrate powders with different phases were investigated, including amorphous and crystalline phases. The powders were directly compressed into compacts at different pressures. The bulk density and mechanical strength of the compacts were measured. The Heckel equation was used to describe the compaction process of the materials. The bonding principles are also discussed based on the microstructure analysis.

## 2. Materials and Methods

### 2.1. Synthesis of Calcium Silicate Hydrate

Calcium silicate hydrate was hydrothermally synthesized by mixing the stoichiometric amounts of calcium oxide (CaO) and siliceous materials (SiO_2_). Calcium oxide with a free CaO content of 88.83% was prepared by calcining lime stone at 800 °C (obtained by Shandong Company, Chongqing). Its specific surface area by Blaine permeability test was 406 m^2^/kg. Nano silica powder and quartz powder were used as siliceous materials. The former was reactive SiO_2_ (AS-200, China BlueStar Shenyang Chemicals, Shenyang, China) with a BET (Brunauer-Emmett-Teller) specific surface area of 181 m^2^/g reported by the producer. It was dried at 105 °C to remove surface adsorbed water before use in hydrothermal synthesis. Quartz powder (99.09% SiO_2_) was obtained by milling α-quartz sand (Yunyang, Chongqing, China). The Blaine fineness was 521 m^2^/kg. 

To obtain different composition of calcium silicate hydrate powders, CaO and SiO_2_ (nano silica powder or α-quartz powder) were mixed in an autoclave (20 L, Weihai Chemical Machinery Co. Ltd., Weihai, China) with Ca/Si molar ratios of 1.0 and 0.83, respectively. Water was then added to the solid mixture with a water/solid weight ratio of 10. The mixture was cured as a stirred suspension (400 rpm) at 120 °C (0.198 MPa steam pressure) and 185 °C (1.1 MPa steam pressure) for 1–24 h. It took 2 h and 4 h to achieve the above curing temperatures of 120 °C and 185 °C, respectively. After hydrothermal synthesis, the suspension was cooled to 50 °C in 20 min before being filtered. The filtered solid product was oven dried to a constant weight at 80 °C, and then dispersed into powder particles with an average size from 10 to 20 μm measured by a light scattering particle size analyzer (Mastersizer 2000). More details about hydrothermal synthesis method are described in [17]. The materials and parameters of hydrothermal synthesis are shown in Table 1. PA was prepared from the mixture of nanosilica powder and calcium oxide, and the other powders (PB to PF) were synthesized from mixtures of quartz and calcium oxide. PR is an α-quartz powder that was used as a reference to compare with the compaction properties of the powders obtained from hydrothermal synthesis. No hydrothermal treatment was applied.

### 2.2. Preparation of Compacts

Prism specimens with the size of 160 × 40 × 40 mm^3^ were prepared by compressing the powders in a die consisting of a prism and two separated pistons. The following procedure was used to prepare the compacts using a compression machine:

- The die was coated with zinc stearate (lubricant) to reduce the friction between the powder and the internal wall of the die.
- The prism was fixed vertically by two steel bearings with the lower piston placed in the prism at approximately 10 mm.
- The oven-dried calcium silicate hydrate powders were added in the prism die, and tamped with a spatula to remove the air from the powder. The amount of C-S-H powder depended on the compaction pressure.
- The upper piston was placed in the prism. Approximately 4.8 kN (0.75 MPa) of pressure was applied to pre-compress the powder before the steel bearings were removed.
- The pre-compressed powder was compressed by increasing the compression pressure to a required value (20, 40, 60 or 80 MPa), with a loading rate of approximately 2.4 kN/s.
- The pressure was kept constant for 3 min after the required value was achieved, followed by quick decompression.

The compacts were thus obtained. To reduce the uncertainty of the results, three specimens were prepared for each series. The bulk density, flexural strength, and compressive strength of the specimen were measured according to ASTM C1639 [18], ASTM C348 [19], and ASTM C349 [20], respectively. Cubic specimens with the size of 50 × 50 × 50 mm^3^ were also prepared using the same method.

### 2.3. Properties of Powders and Compacts

#### 2.3.1. Density

The powder density was determined at room temperature according to ASTM C188 [21]. The results are shown in Table 1. The tamped density, which is the initial bulk density of the powders before compression, was determined according to Ref. [22] using the above compression die. A powder volume of 830 cm^3^ was filled in the die, and tamped with a spatula to remove the air. It was then compressed by the upper piston.

#### 2.3.2. Microstructure Characterization

An X-ray diffractometer, (XRD, RigakuD/max-1200, Osaka, Japan) with Cu Kα radiation of λ = 1.5406 Å, was used to analyze the mineral phases of the powders. A scanning rate of 2°·min^−1^ and step size of 0.02° were used, and the scanning range was from 5° to 65° 2θ. All the samples were stored in a nitrogen atmosphere before measurement. 

Nitrogen gas adsorption was conducted to measure the specific surface area and pore structure of the powders and compacts by using an ASAP 2020. The measurement was carried out at 77 K (Kelvin temperature). Approximately 0.1 g of the sample was used for each measurement. The specific surface area was calculated according to BET multilayer adsorption theory, and the pore structure with a pore diameter from 1.7 nm to 300 nm was determined by BJH (Barrett-Joyner-Halenda) adsorption and desorption isotherm [23].

SEM (Tescan Vega II, Tescan Ltd., Brno, Czech) was applied to detect the morphology and elements of the powders and the fracture sections of the compacts. Its magnification ranged from 4 to 100,000 with a voltage of 20 KV. The instrument was equipped with a secondary electron detector and EDS (Energy dispersive spectroscopy, INCA Energy 3500 X, Oxford, UK). The samples were coated with gold to improve their electrical conductivity before measurement. 

#### 2.3.3. Compaction Behavior of the Powder

The density of the compacts related to the pressures is often used to describe the compaction process of powders, including the particle rearrangement, deformation and/or fragmentation [24]. Many equations have been developed to illustrate powder compaction [25,26]. In this study, the Heckel equation is used to describe the compaction behavior of calcium silicate hydrate powders. The expression is as follows [26]:

(1) $$ln\left(\frac{1}{1-D}\right)=kP+a$$

where *k* (Pa^−1^) is a proportional constant related to the plastic deformation and/or fragmentation of particles, *P* (MPa) denotes the applied compression pressure, and *a* is a constant of densification by particle movement and rearrangement. *D* is a ratio of the bulk density of compacts to the powder density in this study. The term ln[1/(1−D)] represents the densification of the powder during compaction. There is likely nonlinearity in the early stage of compaction due to the effect of particle rearrangement, and *a* is defined as [26]:

(2) $$a=ln\left(\frac{1}{1-D_{0}}\right)+b$$

where *b* is the densification from individual particle movement and rearrangement representing the degree of packing achieved at low pressures before an appreciable amount of interparticulate bonding occurs. *D*_0_ is the ratio of the tamped density to the powder density, and $ln\left[1/\left(1-D_{0}\right)\right]$ is the densification by tamped filling in the die.

## 3. Results

### 3.1. Microstructure of the Powders

#### 3.1.1. XRD Analysis

**Figure 2:**
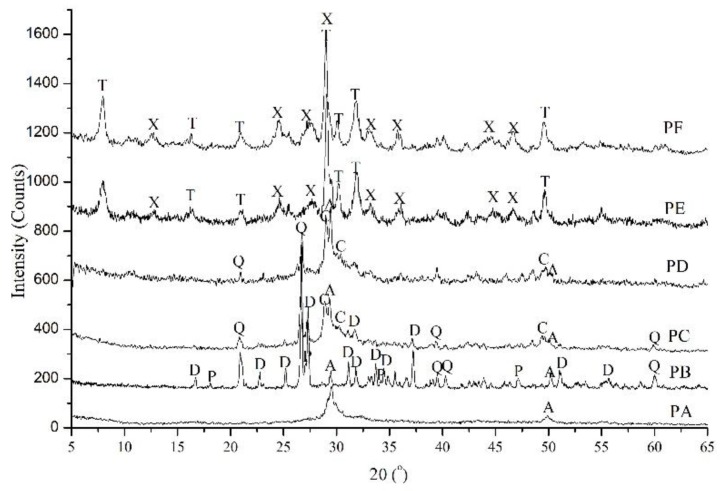
XRD patterns of calcium silicate hydrate powders (PA to PF) with the hydrothermal curing parameters in Table 1. A: Amorphous nature of C-S-H; C: CSH(I); D: α-C_2_SH; Q: α-SiO_2_; P: Ca(OH)_2_; T: Tobermorite; X: Xonotlite.

The XRD patterns of the powders obtained from hydrothermal synthesis (Table 1) display similar peaks at 28°~32° and 50° 2θ (Figure 2), which are typical for calcium silicate hydrates. However, the positions and intensities of these peaks differ; this was attributed to the varied hydrothermal conditions. Only two peaks with *d*-spacings of 0.302 nm (2θ ≈ 29.5°) and 0.183 nm (2θ ≈ 49.9°) were present in PA when nanosilica powder was used in synthesis. These peaks were not distinct, and had a wide background which demonstrates the amorphous nature of calcium silicate hydrate (C-S-H) [2,27]. In contrast, when quartz was used as the siliceous material, the phases in PB were amorphous C-S-H, and crystalline dicalcium silicate hydrate (α-C_2_SH, d-spacing of 0.530, 0.422, 0.390, 0.3237, 0.250, and 0.218 nm) [28,29]. α-SiO_2_ and Ca(OH)_2_ were still present. As the curing temperature increased to 185 °C, In addition to C-S-H, α-C_2_SH and α-SiO_2_, there was more ordered C-S-H with a *d-*spacings of 0.307, 0.280 and 0.183 nm which was named as CSH(I) [30] in PC. PD consisted of amorphous C-S-H, CSH(I) and α-SiO_2_. In PE and PF, the distinct peak at a d-spacing of 1.13 nm (2θ ≈ 7.9°) belongs to well-crystalline tobermorite, and that at 31.8° 2θ is a typical peak for xonotlite. The phase transformation resulting from varied hydrothermal conditions is discussed in [17].

#### 3.1.2. SEM Analysis

**Figure 3:**
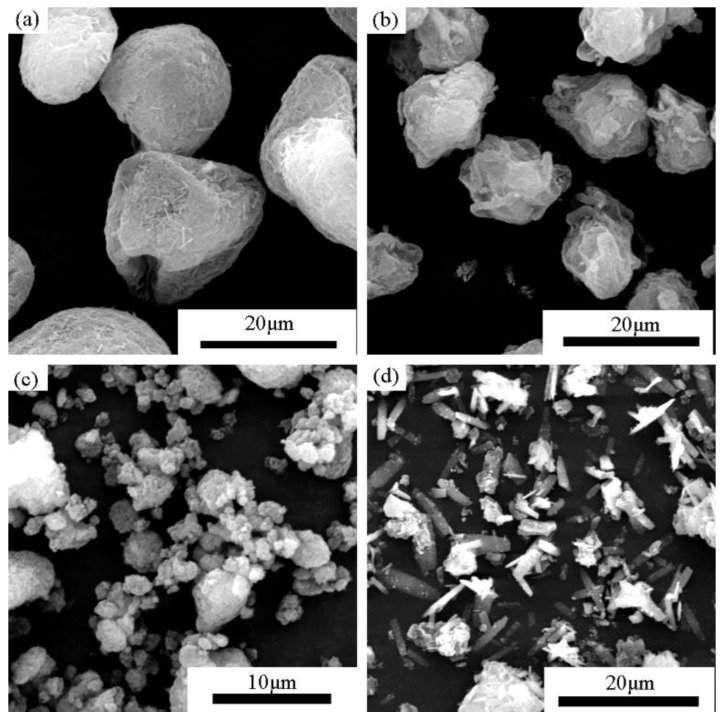
SEM photographs of synthetic C-S-H powders with C/S = 1.0 (**a**) PA, prepared from CaO and nano silica powder at w/s = 10, 120 °C for 10 h (**b**) PB prepared from CaO and α-SiO_2_ at w/s = 10, 120 °C for 10 h; (**c**) PC and (**d**) PE prepared from CaO and α-SiO_2_ at w/s = 10, 185 °C for 1 h and 4 h, respectively.

The morphologies of the synthetic calcium silicate hydrates are shown in Figure 3. Quantitative analysis of the elemental composites was also carried out by point analysis of the EDS data. It can be inferred that agglomeration occurred in PA, PB and PC, which consisted of non-crystallized C-S-H, as shown by the XRD results. The amorphous C-S-H in PA was comprised of microscale fibrils with a length of 2 μm (Figure 3a). In PB, C-S-H with the insertion of rod-like SiO_2_ had an average Ca/Si molar ratio of 0.9 to 1.3 (Figure 3b). Calcium silicate hydrate with reticulated morphology was formed when the mixture was cured at 185 °C for 1 h (Figure 3c). The average Ca/Si molar ratio of this product ranged from 0.97 to 1.08. However, in PE, the product exhibited a typical columnar shape due to the existence of tobermorite and xonotlite. The average Ca/Si molar ratio was 1.175.

### 3.2. Compaction of Calcium Silicate Hydrate Powders

**Figure 4:**
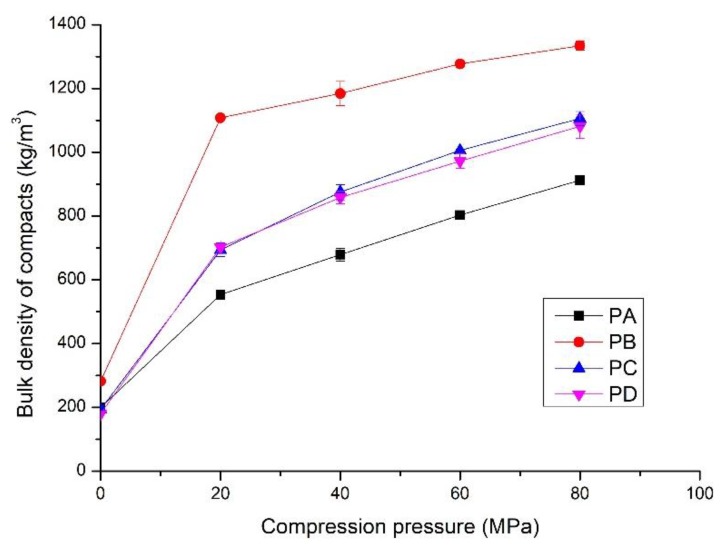
Bulk density of the compacts prepared from PA, PB, PC and PD at different compression pressures; hydrothermal synthesis of the powders shown in Table 1.

**Figure 5:**
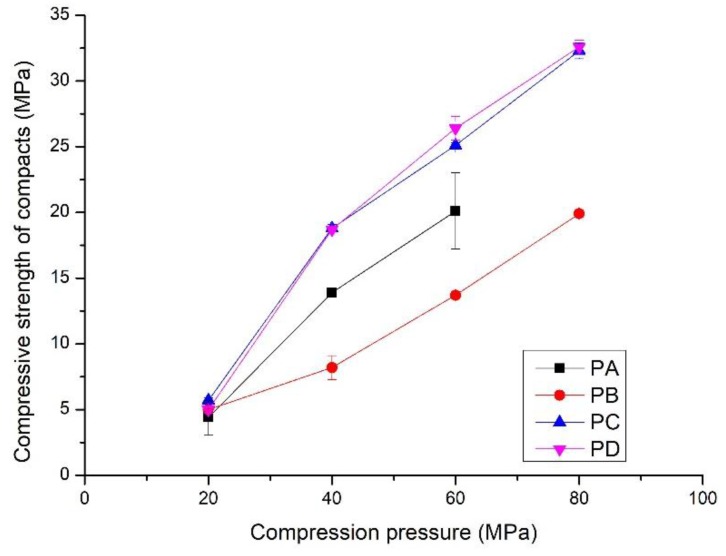
Compressive strength of the compacts prepared by compressing PA, PB, PC and PD at different compression pressures; hydrothermal synthesis of the powders shown in Table 1.

**Figure 6:**
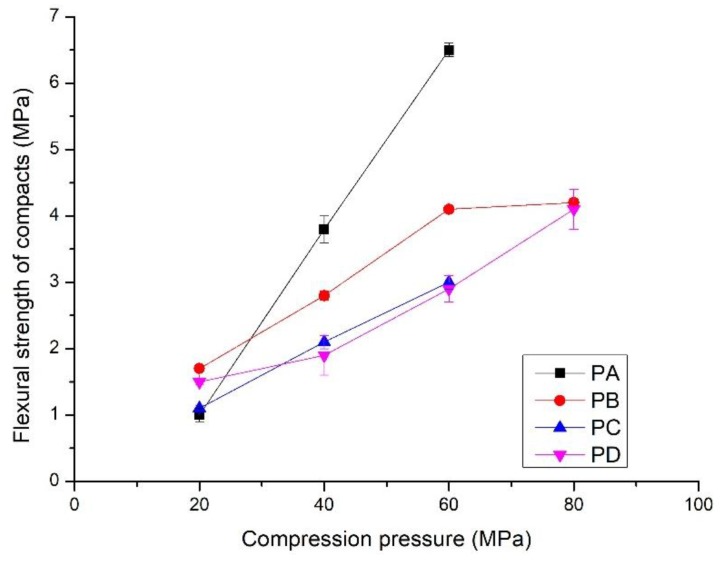
Flexural strength of the compacts prepared by compressing PA, PB, PC and PD at different compression pressures; hydrothermal synthesis of the powders shown in Table 1

This section describes the compacts of the calcium silicate hydrate powders and quartz powder. A preliminary test was conducted to determine the compaction properties of the powders compressed at 40 MPa. The results are illustrated in Table 2. Powders PA to PD could be compressed to form compacted and hardened blocks. The compacts from PA consisted of amorphous phase with the bulk density and compressive strength of 679 kg/m^3^ and 13.9 MPa, respectively. The compacts prepared from PB had the highest bulk density (1184 kg/m^3^) but the lowest compressive strength (8.2 MPa). Powders PC and PD showed similar compaction properties with higher bulk density and compressive strength than the compacts made from PA. Notably, the compacts prepared from PE and PF, which consisted of tobermorite and xonotlite, were severely cracked after being removed from the die, and the α-quartz powder could not be compacted under any of the applied pressures. This primarily resulted from the brittle fracture of the crystallized structure during compaction [16,31]. Therefore, it is inferred that the amorphous C-S-H is necessary for compaction. The bulk density and mechanical properties of the compacts made from PA to PD compressed at 20 to 80 MPa were tested. The results are shown in Figure 4, Figure 5 and Figure 6.

When no compression pressure was applied, the bulk density was the tamped density of the powders, from 200 kg/m^3^ to 300 kg/m^3^ (Figure 4). It increased rapidly as compression pressure increased to 20 MPa, followed by a gradual linear increase with increasing pressure. For the compacts made from PA, the bulk density was 540 kg/m^3^ when the compression pressure was 20 MPa, which is approximately 170% higher than the tamped density. It increased to 912 kg/m^3^ at the compression pressure of 80 MPa. These compacts had the lowest bulk density at all compression pressures due to the high amount of amorphous C-S-H. The compacts compressed from PB showed the highest bulk density, which was 1108 kg/m^3^ and 1334 kg/m^3^ for the compression pressures of 20 MPa and 80 MPa, approximately 105% and 50% higher than those made from PA. This is partly attributed to the high amount of crystalline phases, especially the presence of α-SiO_2_. It could produce a much denser compact due to the filling effect. The compacts prepared from PC and PD at the same compression pressure showed quite similar bulk density, from 500 kg/m^3^ to 1200 kg/m^3^, due to the presence of similar phases. The bulk density of the compacts was between those made from PA and PB at the same compression pressures.

The compressive strength and flexural strength of the compacts increased linearly with compression pressures from 20 to 80 MPa (Figure 5 and Figure 6). Compressive strength varied from 5 to 35 MPa, and the flexural strength increased to 6.5 MPa. Interestingly, the development of strength with the compression pressure differs from that of the bulk density. The compacts made from PB exhibited the lowest compressive strength, from 5 to 20 MPa, although they had the highest bulk density. The compacts compressed from PC and PD showed the highest compressive strength, i.e., from 5.0 to 32.5 MPa. In contrast to the compressive strength, the compacts of PA showed rapid development of the flexural strength (Figure 6). It increased to 6.5 MPa from 1.0 MPa when the compression pressure increased from 20 MPa to. Compacts made from PC and PD had the lowest flexural strength, i.e., from 1.5 to 4.1 MPa. This indicates that the properties of the powders affect the mechanical properties of the compacts. Details are presented in the discussion section.

### 3.3. Morphology of the Compacts

**Figure 7:**
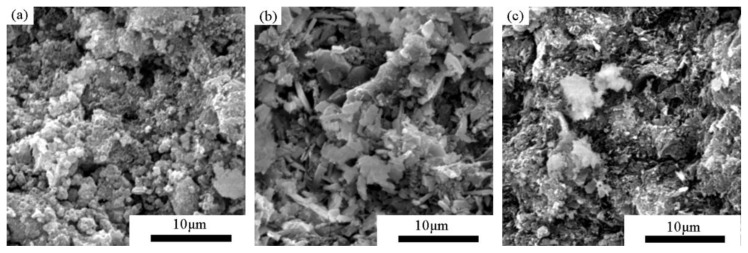
SEM photographs of the specimen by compressing the synthesized powders at 40 MPa: (**a**) compact of PA (the powders synthesized from nano silica and CaO at 120 °C for 10 h with w/s of 10 and Ca/Si of 1), (**b**) compact of PB (prepared from α-quartz and CaO at 120 °C for 10 h with w/s of 10), and (**c**) compact of PC (prepared from quartz and α-CaO at 185 °C for 1 h with w/s of 10).

The morphologies of the compacts prepared by compressing powders PA, PB, and PC at 40 MPa are shown in Figure 6. The microstructures were greatly distinct from those before compression shown in Figure 2. The powder particles generally deformed or fragmented into much smaller sizes. They were subsequently aggregated to form a consolidated structure. The compact made from PA (Figure 7a) had a porous structure and an interface with a gap of approximately 1μm due to the deformation of the powder particles under compression, contributing to a low bulk density. SiO_2_ crystals with a size of 2 to 5 μm were more pronounced in the compacts of PB (Figure 7b) than in the powder before compression (Figure 3b), which was considered the result of fragmentation of the powder particles. The flocculent C-S-H gel and rod-like crystals combined to make the specimen more compact, with a higher bulk density than other specimens. The specimen prepared from Powder C presented a disordered structure (Figure 7c). The interface was also visible, but the particles were much smaller in size.

### 3.4. Pore Structure of the Compacts

**Figure 8:**
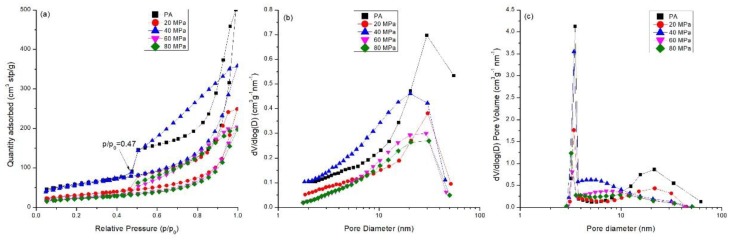
Pore structure of compacts compressed from PA at different compression pressures (20–80 MPa): (**a**) isothermal linear plots; (**b**) pore size distribution based on BJH adsorption isothermal; (**c**) pore size distribution based on BJH desorption isothermal.

Amorphous C-S-H in the powder is essential for producing a compact and its strength development. Consequently, the specific surface area and pore structure of the compacts prepared from amorphous calcium silicate hydrate powder (PA) were investigated (Table 3 and Figure 8). The results in Table 3 show that the *S_BET_* (BET specific surface area) and pore volume of the compacts decreased compared with those of the powder. In addition, the average pore size slightly decreased after compaction calculated either by BJH adsorption isotherm or by BJH desorption isotherm. This indicates that the powder particles aggregated to become a compacted solid. Stanley-Wood and Shubair [32] suggested that the loss of surface area was due to the formation of granule-granule or particle-particle bonds. However, the *S_BET_* of the compacts increased from 109 m^2^/g to 198 m^2^/g when the compression pressure increased from 20 MPa to 40 MPa, which is considered the effect of particle fragmentation. This is in agreement with the SEM analysis.

In Figure 8a, the adsorption curves of powders and compacts are type II, typical of macroporous solids according to IUPAC classification [23]. The very steep part at *p/p*_0_ close to 1 indicates the formation of wide pores attributed to the space between powder particles. Hysteresis loops in all the samples started at the relative pressure *p/p*_0_ = 0.47, a representative of mesopores in the samples. Based on the loop shape, the pores of the powder and compacts were characterized by type H3 [23], suggesting a slit-like pore structure. The amount of gas uptake decreased upon compaction due to the contact of particles narrowing macropores. However, it is noted that the gas amount was higher when the powder was compressed at 40 MPa than at other pressures. This could be ascribed to the formation of mesopores during particle fragmentation by compression. As a result, unimodal distribution of the powder and compacts was present based on adsorption isothermal branches. The most probable pore size appeared at approximately 30 nm for PA and compressed at 20 MPa, 60 MPa, and 80 MPa, and the value decreased to 20.2 nm at 40 MPa (Figure 8b). The pore size distribution was different when calculated based on BJH desorption isotherm (Figure 8c) due to the hysteresis loops which were closed at *p/p*_0_ = 0.47. The pore size between 3 and 4 nm was pronounced. Specifically, the pore size distribution of compacts compressed at 20 MPa was similar to that of PA, and both have a bimodal distribution with the most probable pore sizes at approximately 3.5 and 21.6 nm. As the compression pressure increased to 40~80 MPa, the bimodal distribution of pores changed into unimodal distribution, with larger pores gradually disappearing; this was attributed to the collapse of the macropores by compaction. At 40 MPa, the number of larger pores decreased. Instead, the number of mesopores smaller than 10 nm increased, with the most probable pores appearing at 3.4 nm. This demonstrates the fragmentation of larger particles into smaller ones, which fill in the voids and refine the pore size. The most probable pore size decreased with increasing compression pressures. The values were 3.3 nm at 60 MPa and 3.2 nm at 80 MPa, indicating a coherent and compacted structure gradually formed with an increasing compression pressure. 

## 4. Discussion

### 4.1. Compaction Process

In this section, the Heckel equation (Equations (1) and (2)) is used to study the compaction process of the calcium silicate hydrate powders. Plots obtained from the relationship between the bulk density of compacts and compression pressures (Figure 3) are shown in Figure 8. It has been reported that the increase in the bulk density of compacts with compression pressure is related to particle rearrangement, particle fragmentation, and/or plastic and elastic deformation [26,33]. The former two stages are involved in the oven-dried calcium silicate hydrate samples. The elastic deformation can be ignored because of the removal of some physically adsorbed water [34].

**Figure 9:**
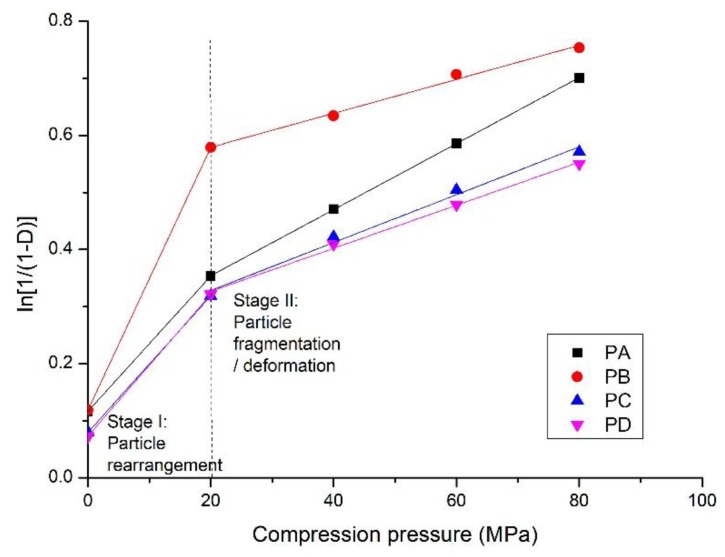
The Heckel plots of compaction of the calcium silicate hydrate powders shown in Table 1.

At stage I, the densification ln[1/(1−D)] initially increases rapidly. It often occurs at a relatively lower compression pressure, for instance, at 20 MPa where a knee point appears, as shown in Figure 9. The densification is primarily due to the rearrangement of the powder particles, as finer particles penetrate into the voids between the larger ones resulting in a closer packing arrangement. This leads to a decrease in the specific surface area and pore volume, as shown in Table 3. The powder particles behave individually rather than a coherent mass, which is indicated by the similar pore size distribution of the powder and the compacts prepared at a compression pressure of 20 MPa (Figure 8). In this stage, point contacts are dominant and few interparticulate bonding sites are formed, resulting in a relatively lower strength. SEM analysis on the compacts of calcium silicate hydrate powder from fly ash also demonstrate the rearrangement of particles at the compression pressures lower than 40 MPa (Figures 3 and 6 in [35]).

The subsequent increase (stage II) in densification is accompanied by plastic deformation and/or fragmentation of the particles, as shown by a linear plot of ln[1/(1−D)] with compression pressure from 20 MPa to 80 MPa, forming a coherent structure. According to the N_2_ adsorption analysis *(*Section 3.4), the fragmentation of particles mainly occurs at compression pressures from 20 to 40 MPa, as the specific surface area and pore volume increase with increasing pressure. The large particles are fragmented into smaller ones by compression, and fill in the voids to make the structure more compacted with a smaller average pore size. The SEM measurement demonstrated that the particle size was much smaller when compressed at 40 MPa (Figure 7) than before compression (Figure 2). Structure deformation, particularly the sliding of sheets one onto the others in the solid C-S-H particles also occurs under high pressures/stress as reported previously [36,37]. The N_2_ adsorption measurement shows that the pore structure of the compacts prepared at 60 MPa and 80 MPa was nearly the same, suggesting that plastic deformation of the particles is dominant at a higher compression pressure. In this stage, more contact sites are created to improve the mechanical strength.

The values of constants *a* and *b* from Equations (1) and (2) shown in Table 4 indicate the compaction properties of the powders. The slope *k* reflects the plasticity and 1/*k*, known as the yield pressure, is associated with particle deformation. A higher slope (*k*) indicates higher plastic deformation [31,38]. The constant *a* represents the particle movement at low pressures before interparticulate bonding occurs, and *b* denotes that the powders have a certain densification by individual particle movement in Stage I, with a higher value for higher compressibility [39]. PA has the highest plastic deformation, which is attributed to the amorphous C-S-H, and PB is the most compressible material in this study because of the lowest specific surface area and the presence of crystalline phases. The potential for the compaction process depends on the nature of the powders, including their phases.

### 4.2. Bonding Principles on the Compaction of the Powders

**Figure 10:**
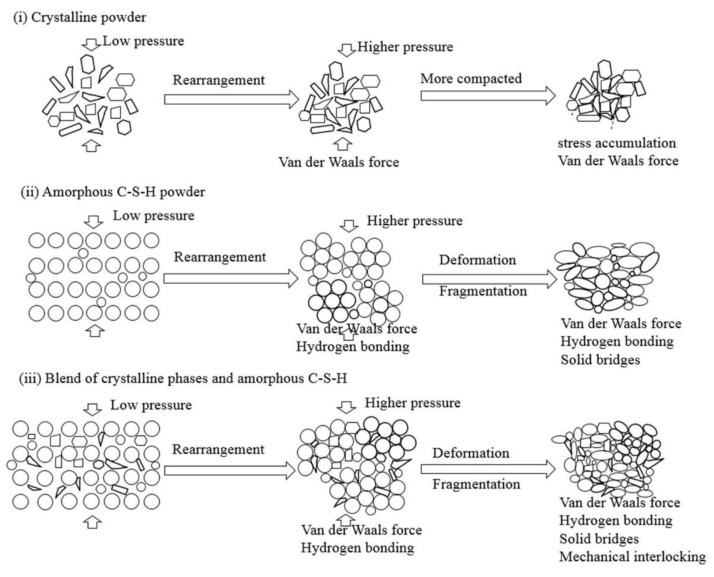
Schematic diagram illustrating the bonding of different types of powder particles during compaction (**i**) crystalline powders; (**ii**) amorphous C-S-H powder; (**iii**) blend of crystalline phases and amorphous C-S-H.

The microstructure analysis and mechanical properties of the compacts show that the powders with amorphous C-S-H have contact-hardening properties. Crystalline calcium silicate hydrate and α-SiO_2_ powders are difficult to compact and/or harden, as shown in Table 2. This suggests that amorphous C-S-H plays a key role in producing strong bonds during compression for developing the strength of the compacts. Stemmermann et al. [13] considered the ionic bond as a predominant factor in strength development. Glukhovsky [16] assumed that the bond formed by point contact of particles greatly improves the strength of compacts. In this section, different bonding patterns during compaction that are related to the properties of amorphous and crystalline C-S-H are discussed and are shown in the schematic diagram in Figure 10.

Generally, solid bridges, interparticulate bonding (Van der Waals forces and hydrogen bonds), and mechanical interlocking are dominant in the consolidation of dried powders. The most important factors to the bonding strength are the bonding surface area and interparticulate bonding across particle-particle interfaces [40]. Well crystallized powders (Figure 10i), such as quartz powder, are difficult to compact and harden at the applied pressures. This is because very few interparticulate linkages are formed and the bond is van der Waals forces, which result in the weak bonding strength of the compacts [41]. Schneider et al. [42] note that a large contact surface area between quartz particles could be created only when the grain size decreases to approximately 80 nm by shock loading at high pressure, e.g., 500 MPa. In addition, considerable stress concentrated along the edges of the crystals during compression may lead to cracks in the compacts, such as in the compaction patterns of PE and PF (Table 2).

Different from the well-crystallized powders, amorphous C-S-H, which is a layered silicate structure with a certain amount of adsorbed water and chemical combined water, has strong interparticulate attractions such as cohesion forces between the faces of C-S-H particles [43]. Due to the rearrangement of the powder particles at low compression pressure, the distance between particles is greatly reduced, the particles have close contact, and a small number of contact points are formed. The dominant interparticulate attractions are van der Waal’s forces and hydrogen bonds (Figure 10ii) formed at the distance of 100~1000 Å [24]. This results in the relatively low bulk density and mechanical strength of the compacts prepared from PA at 20 MPa (Figure 4, Figure 5 and Figure 6). With increasing compression pressure, the point contact is transformed to surface contact, which is attributed to the deformation and/or fragmentation of the particles. A small number of solid bridges can form at higher compression pressures when the particle-particle contact is at an atomic level, which is usually considered a continuous phase of powder material [24,44]. These bridges develop where there is a certain degree of plastic deformation of the powder particles at a high concentration of stress levels. This can be estimated from the surface specific strength of the compacts which is a ratio of the strength to the surface area of the material suggested by Nyström et al. [45]. As shown by the flexural strength (Figure 5) and specific surface area of the compacts (Figure 8), the surface specific flexural strength was 0.044 N/m^2^ when PA was compressed at 40 MPa, slightly higher than that of the compacts at the compression pressure of 20 MPa (0.026 N/m^2^). In this case, van der Waals forces and hydrogen bonds are still dominant. The value increased to 0.136 N/m^2^ when the powder was compressed at 60 MPa. The dramatic increase in the surface specific flexural strength indicates the formation of solid bridges resulting in strong particle-particle bonds [44,45]. This is likely because the oxygen atom of water (Ow) and the hydrogen atom in the C-S-H structure can be easily diffused at the atomic level [46]. For example, the atoms can be transformed between water molecules and Si-OH, Ca-OH groups, which influences the connectivity of atoms in C-S-H structure and affects the bonding strength of the C-S-H particles.

For the powders blended with amorphous and crystalline phases (PB to PD, etc.), in Figure 10iii, van der Waals forces and hydrogen bonds are dominant, as shown by the mechanical strength of the compacts compressed at low compression pressure. This is indicated by the similar strength of the compacts made from different powders at 20 MPa (Figure 4 and Figure 5). When the compression pressure increases, the bonding types differ from those of the amorphous C-S-H powder compacts. Mechanical interlocking, resulting from the adhesion between C-S-H particles and the crystalline phases [47,48], occurs when there is surface contact. This may have a positive effect on the compressive strength as the compacts prepared from PC and PD show higher strength than those made from PA at the same compression pressure. However, the bonding strength is weakened if too many crystalline phases are present in the powder, as demonstrated by the lower compressive strength of compacts from PB. Notably, the types of bonding affect the compressive strength and flexural strength of the compacts.

## 5. Conclusions

The results reveal that powders containing amorphous C-S-H can be used to produce hardened compacts. The compressive strength of the compacts is from 5 MPa to 32 MPa with a bulk density from 500 kg/m^3^ to 1340 kg/m^3^. The tobermorite, xonotlite, and α-quartz powder with well crystallized structure could not be compacted or hardened. This shows that the amorphous C-S-H plays a decisive role in the development of strength by compression. The formation of interparticulate attractions including Van der Waals forces and hydrogen bonding is significant for strength development at low compression pressures. Solid bridges and mechanical interlocking can occur at high compression pressures, resulting in improvement of the bonding strength and mechanical properties of the compacts.

The compaction process occurs into two stages, which are the rearrangement of powder particles and the fragmentation and/or deformation of C-S-H particles. The former stage is associated with a considerable increase in bulk density and a substantial decrease in specific surface area, which usually occurs at a low compression pressure. Point contact is dominant in this stage, leading to the relatively low strength of the compacts. As the compression pressure increases, powder particles become fragmented and/or deformed. This results in the gradual increase in the bulk density of the compacts. Point contact is transformed to surface contact, leading to more bonds, and consequently, to increased strength. The results also demonstrate that the contact- hardening of compacts is influenced by the crystalline nature of the powders.

## Figures and Tables

**Table 1:** Hydrothermal synthesis parameters of calcium silicate hydrate.

Index of Powder	Materials		Ca/Si (Molar Ratio)	Water/Solid (w/s, by Weight)	Hydrothermal Curing		Powder Density (kg/m^3^)
	Siliceous Material	Calcareous Material			Temperature (°C)	Time (h)	
PA	Nanosilica	Calcium oxide	1.0	10	120	10	1810
PB	α-quartz	Calcium oxide	1.0	10	120	10	2520
PC	α-quartz	Calcium oxide	1.0	10	185	1	2540
PD	α-quartz	Calcium oxide	1.0	10	185	2	2554
PE	α-quartz	Calcium oxide	1.0	10	185	4	2381
PF	α-quartz	Calcium oxide	0.83	10	185	24	2660
PR	α-quartz	/	/	/	/	/	2670

**Table 2:** Compaction patterns of calcium silicate hydrate powders and quartz powder at a compression pressure of 40 MPa.

Index of Powder	Visual of the Specimen		Bulk Density of the Specimen (kg/m^3^)	Compressive Strength (MPa)
PA	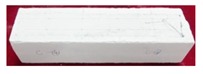	Perfect	679	13.9
PB	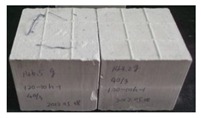	Some defects along the edge	1184	8.2
PC	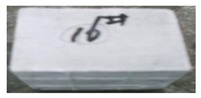	Perfect	875	18.8
PD	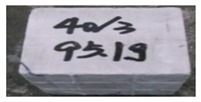	Perfect	858	19.2
PE	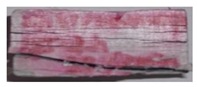	Cracks	Failed to measure	Failed to measure
PF	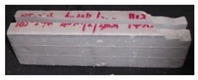	Separated	Failed to measure	Failed to measure
α-Quartz powder	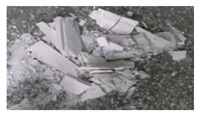	Unable to be compacted	Failed to measure	Failed to measure

**Table 3:** Specific surface area and pore structure of compacts obtained from PA at different pressures (20–80 MPa).

Compression Pressure		No Pressure (PA)	20 MPa	40 MPa	60 MPa	80 MPa
Specific surface area, *S_BET_* (m^2^/g)		204	109	198	83	78
Pore volume (cm^3^/g)		0.78	0.39	0.56	0.31	0.31
Average pore size (nm)	BJH adsorption	16.1	13.5	10.8	13.2	13.5
	BJH desorption	9.1	7.7	5.7	6.8	6.3

**Table 4:** Values of constants from Equations (1) and (2) for the compacts of synthesized calcium silicate hydrate powders shown in Table 1.

Constant	PA	PB	PC	PD
*a*	0.24	0.52	0.25	0.26
*k* (Pa^−1^)	5.79 × 10^−9^	2.98 × 10^−9^	4.06 × 10^−9^	3.62 × 10^−9^
*b*	0.12	0.40	0.17	0.19
$\ln\left[1/\left(1-D_{0}\right)\right]$	0.116	1.119	0.080	0.073

## References

[1] Nonat A. (2004). The structure and stoichiometry of C–S–H. Cem. Concr. Res..

[2] Taylor H.F.W. (1997). Cement Chemistry.

[3] Pellenq R.J.M., Lequeux N., van Damme H. (2008). Engineering the bonding scheme in C–S–H: The iono-covalent framework. Cem. Concr. Res..

[4] Wang S., Peng X., Zeng L. (2017). Research on contact-hardening cementitious properties of Nano-amorphous calcium silicate hydrate. J. Hunan Univ. Nat. Sci..

[5] Taylor H.F.W. (1986). Proposed Structure for Calcium Silicate Hydrate Gel. J. Am. Ceram. Soc..

[6] Alderborn G., Nyuström C. (1995). Pharmaceutical Powder Compaction Technology.

[7] Karamchandani A., Yi H., Puri V.M. (2016). Comparison and explanation of predictive capability of pellet quality metrics based on fundamental mechanical properties of ground willow and switchgrass. Adv. Powder Technol..

[8] Brewin P.R., Coube O., Doremus P., Tweed J.H. (2008). Modelling of Powder Die Compaction.

[9] Sereda P.J., Feldman R.F. (1963). Compacts of powdered material as porous bodies for use in sorption studies. J. Appl. Chem..

[10] Feldman R.F., Beaudoin J.J. (1976). Microstructure and strength of hydrated cement. Cem. Concr. Res..

[11] Beaudoin J.J. (1983). Comparison of mechanical properties of compacted calcium hydroxide and portland cement paste systems. Cem. Concr. Res..

[12] Jennings H.M., Hodson S.K. (1997). Compressed Low Density Hydraylically Bonded Composite Articles.

[13] Stemmermann P., Garbev K., Beuchle G., Schweike U. (2010). Method for Producing Components.

[14] Lin W., Zhang C., Fu J., Xin H. (2018). Dynamic mechanical behaviors of calcium silicate hydrate under shock compression loading using molecular dynamics simulation. J. Non-Cryst. Solids.

[15] Wang S., Peng X., Tao Z., Tang L., Zeng L. (2017). Influence of drying conditions on the contact-hardening behaviours of calcium silicate hydrate powder. Constr. Build. Mater..

[16] Glukhovsky V.D., Runova Р.F., Маxcunov С.Е. (2004). Contact-Hardening Cementitious Materials and Compounds.

[17] Wang S., Peng X., Tang L., Zeng L., Lan C. (2018). Influence of hydrothermal synthesis conditions on the formation of calcium silicate hydrates: From amorphous to crystalline phases. J. Wuhan Univ. Technol. Mater. Sci. Ed..

[18] ASTM (2017). ASTM C1693. Standard Specification for Autoclaved Aerated Concrete (AAC).

[19] ASTM (2014). ASTM C348-14. Standard Test Method for Flexural Strength of Hydraulic-Cement Mortars.

[20] ASTM (2014). ASTM C349-14. Standard Test Method for Compressive Strength of Hydraulic-Cement Mortars (Using Portions of Prisms Broken in Flexure).

[21] ASTM (2017). ASTM C188. Standard Test Method for Relative Density of Hydraulic Cement.

[22] USP (2012). Bulk Density and Tapped Density of Powders.

[23] Kruk M., Jaroniec M. (2001). Gas adsorption characterization of ordered organic-inorganic nanocomposite materials. Chem. Mater..

[24] Nyström C., Karehill P.-G., Alderborn G., Nyström C. (1995). The importance of intermolecular bonding forces and the concept of bonding surface area. Pharmaceutical Powder Compaction Technology.

[25] Çomoğlu T. (2007). An overview of compaction equations. J. Fac. Pharm. Ank..

[26] Heckel R.W. (1961). Density-pressure relationship in powder compaction. Trans. Metall. Soc. AIME.

[27] Russias J., Frizon F., Cau-Dit-Coumes C., Malchère A., Douillard T., Joussot-Dubien C. (2008). Incorporation of Aluminum into C–S–H Structures: From Synthesis to Nanostructural Characterization. J. Am. Ceram. Soc..

[28] Yang N.R., Yue W.H. (2000). Handbook of Spectroscopy of Inorganic Nonmetallic Materials.

[29] NocuÒ-Wczelik W. (1999). Effect of Na and Al on the phase composition and morphology of autoclaved calcium silicate hydrates. Cem. Concr. Res..

[30] Luke K. (2004). Phase studies of pozzolanic stabilized calcium silicate hydrates at 180 °C. Cem. Concr. Res..

[31] Schmidt P.C., Herzog R. (1993). Calcium phosphates in pharmaceutical tableting. Pharm. World Sci..

[32] Stanley-Wood N.G., Shubair M.S. (1980). The variation of the surface topography of granules under compression with degree of binder addition. Powder Technol..

[33] Linse V.D., Bruggeman G., Weiss V. (1985). Dynamic compaction of metal and ceramic powders. Innovations in Materials Processing.

[34] Feldman R.F. (1972). Mechanism of creep of hydrated portland cement paste. Cem. Concr. Res..

[35] Wang S., Peng X., Lan C., Tang L. High-strength light weight blocks prepared from the by-product of aluminium removal from fly ash. Proceedings of the International Symopsium EcoCrete Iceland 2014 on Sustainablity, Environmental Friendly Concrete.

[36] Sanahuja J., Dormieux L. (2010). Creep of a C–S–H gel: Micromechanical approach. Int. J. Multiscale Comput. Eng..

[37] Tamtsia B.T., Beaudoin J.J. (2000). Basic creep of hardened cement paste A re-examination of the role of water. Cem. Concr. Res..

[38] Duberg M., Nyström C. (1986). Studies on direct compression of tablets XVII. Porosity—Pressure curves for the characterization of volume reduction mechanisms in powder compression. Powder Technol..

[39] Buckner I.S. (2008). Compression Calorimetry, Powder Compaction Thermodynamics and Deformation Mechanisms. Ph.D. Thesis.

[40] Buckton G., Celik M. (2011). Intermolecular bonding forces: Where materials and process come togther. Pharmaceutical Powder Compaction Technology.

[41] Führer C., Alderborn G., Nyström C. (1995). Interparticulate attraction mechanisms. Pharmaceutical Powder Compaction Technology.

[42] Schneider H., Vasudevan R., Hornemann U. (1984). Deformation of experimentally shock-loaded quartz powders: X-ray line broadening studies. Phys. Chem. Miner..

[43] Dharmawardhana C.C., Misra A., Aryal S., Rulis P., Ching W.Y. (2013). Role of interatomic bonding in the mechanical anisotropy and interlayer cohesion of CSH crystals. Cem. Concr. Res..

[44] Mattsson S. (2000). Pharmaceutical Binders and Their Function in Directly Compressed Tablets—Mechanistic Studies on the Effect of Dry Binders on Mechnical Strength, Pore Structure and Disintegration of Tablets.

[45] Nyström C., Alderborn G., Duberg M., Karehill P.-G. (1993). Bonding Surface area and Bonding Mechanism-Two Important Factors for the Understanding of Powder Comparability. Drug Dev. Ind. Pharm..

[46] Hou D., Zhao T., Jin Z., Li Z. (2015). Structure, reactivity and mechanical properties of water ultra-confined in the ordered crystal: A case study of jennite. Microporous Mesoporous Mater..

[47] Tabor D., Kreijger P. (1981). Principles of Adhesion—Bonding in Cement and Concrete. Adhesion Problems in the Recycling of Concrete.

[48] Kinloch A.J. (1987). Adhesion and Adhesives Science and Technology.

